# The power of language-concordant care: a call to action for medical schools

**DOI:** 10.1186/s12909-019-1807-4

**Published:** 2019-11-06

**Authors:** Rose L. Molina, Jennifer Kasper

**Affiliations:** 1 Division of Global and Community Health, Department of Obstetrics and Gynecology, Beth Israel Deaconess Medical Center, Boston, MA 02215 USA; 2 Scholars in Medicine Office, Harvard Medical School, Boston, 02115 MA USA; 3 Pediatrics and Global Health and Social Medicine, Harvard Medical School, Boston, MA 02115 USA; 4 Department of Newborn Medicine, Brigham and Women’s Hospital, Boston, MA 02115 USA

**Keywords:** Language-concordant care, Health equity, Medical school, Language skills

## Abstract

We live in a world of incredible linguistic diversity; nearly 7000 languages are spoken globally and at least 350 are spoken in the United States. Language-concordant care enhances trust between patients and physicians, optimizes health outcomes, and advances health equity for diverse populations. However, historical and contemporary trauma have impaired trust between communities of color, including immigrants with limited English proficiency, and physicians in the U.S. Threats to informed consent among patients with limited English proficiency persist today. Language concordance has been shown to improve care and serves as a window to broader social determinants of health that disproportionately yield worse health outcomes among patients with limited English proficiency. Language concordance is also relevant for medical students engaged in health care around the world. Global health experiences among medical and dental students have quadrupled in the last 30 years. Yet, language proficiency and skills to address cultural aspects of clinical care, research and education are lacking in pre-departure trainings. We call on medical schools to increase opportunities for medical language courses and integrate them into the curriculum with evidence-based teaching strategies, content about health equity, and standardized language assessments. The languages offered should reflect the needs of the patient population both where the medical school is located and where the school is engaged globally. Key content areas should include how to conduct a history and physical exam; relevant health inequities that commonly affect patients who speak different languages; cultural sensitivity and humility, particularly around beliefs and practices that affect health and wellbeing; and how to work in language-discordant encounters with interpreters and other modalities. Rigorous language assessment is necessary to ensure equity in communication before allowing students or physicians to use their language skills in clinical encounters. Lastly, global health activities in medical schools should assess for language needs and competency prior to departure. By professionalizing language competency in medical schools, we can improve patients’ trust in individual physicians and the profession as a whole; improve patient safety and health outcomes; and advance health equity for those we care for and collaborate with in the U.S. and around the world.

## Background

This article argues for the importance of language-concordant care (clinical encounters in which the patient and care provider speak the same native language) and the role of medical schools in providing language courses to support medical students in delivering language-concordant care. While the ethics around providing meaningful access to language services for all patients through qualified interpreters are critical, we advocate for language courses to improve language competency in clinical encounters and appropriate language assessments of providers who identify as being proficient in multiple languages.

## Main text

“In this work against sickness, we begin not with genetic or cellular interactions, but with human ones. They are what make medicine so complex and fascinating. How each interaction is negotiated can determine whether a doctor is trusted, whether a patient is heard, whether the right diagnosis is made, the right treatment given. But in this realm, there are no perfect formulas.” [1] (p65)

If we could be granted a superpower, many of us would choose omnilingualism—the ability to speak, read, and write all languages. We live in a world of incredible linguistic diversity; nearly 7000 languages are spoken globally and at least 350 are spoken in the United States. Globally, the five most spoken languages are Chinese, English, Hindi, Spanish and Arabic; an estimated 650 million speak English as a second language [2]. According to 2016 U.S. Census data, 25.4 million Americans speak English less than “very well” [3] and the Bureau expects this to remain relatively constant in 2020 [4]. As the U.S. and other countries around the world become more ethnically and culturally diverse, the power to communicate and interact with each other holds even greater importance [5].

In the U.S., language concordance has been vaguely defined as a clinical encounter where the patient and doctor speak the same non-English language [6]. In countries where there is more than one dominant language, such as Canada, scholars have defined language concordance as situations where health care providers and patients speak to each other in their shared native language [7]. Language-discordant encounters occur when patients and health care providers speak different first languages, which may manifest as differences in proficiency and experience and therefore hinder the ability to communicate nuances critical for understanding [7]. Language concordance is a particularly important foundation to gain trust, optimize health outcomes and advance health equity in diverse patient populations. Medical and other health professional schools [8] are optimally positioned to catalyze a deeper sensitivity to language and professionalize language curricula to cultivate skills and behaviors in students who strive for language concordance in clinical encounters. While this debate focuses on literature in the U.S., the challenge of language concordance in clinical care is relevant around the world [5].

The phenomenon of trust and its essential role in human cooperation and competition was investigated experimentally for the first time in the 1950s [9]. Trust is defined as “a judgement by the trustor [e.g. patient], requiring the acceptance of resultant vulnerability and risk, that the trustee (individual or organisation, [e.g. physician]) has the competence, willingness, integrity and capacity (i.e. trustworthiness) to perform a specified task under particular conditions.” [10] Applying Deutsch’s theory of cooperation and competition to the patient-clinician therapeutic alliance, the process needs to include honesty, confidence, and open communication. Patients’ health outcomes depend on their belief that they share valued goals with their physicians. An inability to articulate valued goals or a breach in trust because of a language barrier hence becomes an obstacle to developing a therapeutic alliance and improving health outcomes.

Historical trauma has broken trust between communities of color, including immigrants with limited English proficiency, and physicians. One example is the forced sterilization of Mexican women in Los Angeles county in the late 1960s–1970s. In *Madrigal v. Quilligan*, working-class migrant women reported they underwent tubal ligation at the time of their cesarean deliveries without their informed consent [10]. While the plaintiffs lost the case, they won important changes to informed consent around sterilization, including the requirement of bilingual consent forms [11].

Threats to informed consent among patients with limited English proficiency persist today. A retrospective review of patients undergoing lumbar puncture, thoracentesis or paracentesis at a hospital with interpreter services found English-speaking patients were almost twice as likely to have full informed consent documentation as those with limited English proficiency [12]. Notably, fewer than half of patients with limited English proficiency had a consent form in their primary language or an English consent form signed by an interpreter [12]. In addition to communication challenges in obtaining informed consent, a systematic review demonstrated that language discordance between patients and physicians diminished patients’ sense of integrity and intensified their vulnerability in the clinical setting [13].

In addition to the accurate exchange of information necessary to ensure patient safety, language concordance builds trust in the patient-doctor relationship. While interpreters play important roles in clinical encounters, language-concordant providers are better equipped to build rapport and bond with patients, provide higher quality care, and increase patient satisfaction than language-discordant providers with in-person or telephone-based interpreters [6]. A study of pediatric patients presenting to an urban emergency room found that, in comparison to English-speakers, Spanish-speaking patients had lower trust of individual physicians and higher mistrust of the medical care they receive in comparison to other ethnic groups [14]. In addition, language was a driver of health disparities; Spanish speakers had lower intervention and hospital admission rates [14]. Meeting a doctor who speaks the same language puts patients at ease by alleviating their anxiety about communication; this fosters a sense of relief and gratitude, which form the basis for a therapeutic alliance.

Language concordance between physicians and patients has been shown to improve care through fewer medical errors, increased understanding of illness and the treatment plan, adherence to the treatment plan, and satisfaction with care [15]. In one observational study of Latinx patients with diabetes, patients were asked to describe how well their personal physician spoke their preferred language without an interpreter. Language-discordant physicians were those who patients reported only spoke “fair,” “poorly,” or “does not speak my language.” By contrast, language-concordant physicians were those who patients reported as speaking the preferred language “well,” “very well,” or “excellent.” The study demonstrated that patients with limited English proficiency who had a language-discordant physician had higher odds of poor glycemic control compared to those who had a language-concordant physician, even after controlling for potential demographic and clinical confounders [16]. Communicating health-related information in the appropriate language and literacy level is critical to ensure understanding of and ability to complete treatment plans, and thereby optimize health outcomes [17, 18].

Language-concordant care also serves as a window into understanding the broader social determinants of health that disproportionately yield worse health outcomes among patients with limited English proficiency. In the United States, the Patient Protection and Affordable Care Act of 2010 created Accountable Care Organizations (ACOs) and Health Communities (AHCs) to respond to the burgeoning evidence that addressing health-related social needs through enhanced clinical encounters and clinical-community liaisons can improve health outcomes and reduce costs [19, 20]. Through culturally humble and linguistically appropriate communication, physicians are positioned to discuss sensitive topics such as food and housing insecurity and intimate partner violence; ask about barriers that impede their patients’ health; and facilitate connecting their patients to needed resources. Language alone can be an insurmountable barrier that makes navigating other social determinants of health—such as access to safe housing, job security, and transportation—nearly impossible. Providing language-concordant care is an opportunity for physicians and care teams to help patients overcome the social factors that impact their health and well-being [21].

In addition to the clinical encounters in the United States, language fluency and humility is relevant for medical students engaged in healthcare around the world. Global health experiences among U.S. medical and dental students have quadrupled in the last 30 years [22, 23]. One-third of graduating medical students report a global health experience. Yet, appropriate training in language proficiency and skills to address cultural aspects of clinical care, research and education are lacking in pre-departure trainings [24]. Rather, most trainings focus primarily on safety and travel logistics, and a much smaller proportion address ethics and cultural humility. A web-based review of U.S. medical schools found that 32 (24%) had a structured global health program, but only one addressed language or cultural proficiency [25]. A recent study of eight undergraduate global health programs found only one that explicitly named language proficiency training as a key course objective [26]. Linguistic disparities can negatively impact research, education, patient care, capacity-building and system strengthening in global settings [27–29]. In response, the World Health Organization (WHO) is leading efforts to bridge language gaps in public health [2]. One example is the “Voices in Global Health” course offered at Duke Global Health Institute, where students speak in a non-English language while learning key global health topics [30]. Given the critical interplay of language, culture, trust, collaboration and ethics in global health settings, now is an opportune time to introduce and enhance language awareness and training in global health courses offered during undergraduate medical education.

To develop a workforce with competency in language-concordant care, medical schools will need to integrate language courses into the curriculum and provide opportunities for students to maintain and improve their skills as they learn clinical medicine. Currently, there is a relative paucity of options, with the majority of schools focused only on Spanish courses. A national survey of medical schools found that 66% had a medical Spanish curriculum in place in 2012–2014, and 62% of those schools reported the medical Spanish curriculum had been in place for at least five years [31]. Additionally, 32% of medical schools without a Spanish curriculum planned to implement one in the subsequent two years [31]. The most common reasons cited for implementing this type of curriculum were the growing Spanish-speaking patient population and student interest. A variety of teaching strategies were employed, such as didactic instruction, student-to-student role play, standardized patients, and clinical encounters with patients [31]. Additionally, some institutions integrated interpreter shadowing, online modules, and case discussions. However, there was marked variation in the types of assessments done at the end of the courses: few schools used an Objective Structured Clinical Examination or standardized patient evaluation, and only one used a formal language competency assessment from an external company [31].

While these findings demonstrate an interest in providing language courses during medical training, work must be done to professionalize language competency in medical schools before allowing students to use their language skills in clinical encounters. Some recommendations include setting fluency level criteria for curricula and developing modules that can facilitate retention of language skills over time [7]. In fact, the Affordable Care Act Rule 1557 strictly regulates “meaningful access” for patients with limited English proficiency, including qualifications for those who can act as interpreters. Some hospitals have begun to implement language competency assessments for health professionals who self-identify as being proficient in another language. To achieve equity in communication, health professionals need to demonstrate competency in communicating nearly as well in another language as they do in their native language. However, linguistic competency is not the only *sine qua non* of the patient – provider relationship [32]; other aspects of building a therapeutic alliance should also be intentionally taught and role-modeled in medical education.

Given the complexity of language barriers spanning from linguisitic proficiency to health literacy to body language and other aspects of communication, it is critical for medical schools to develop curricula through interdisciplinary collaboration with experts in health communication science, linguistics, and social science. To operationalize language concordance in medical education training, feasibility challenges and unintended consequences may arise. Leadership support, financial sustainability, and student interest are critical for success. Additionally, language curricula and competency assessments should be developed with the goal of benchmarking a level of proficiency at which physicians can be trusted to practice safely in that language. Without such an evaluation, graduates may feel ready to use their acquired language skills in clinical encounters despite lacking sufficient competency to communicate effectively. For example, international medical graduates need to demonstrate a level of language proficiency as a pre-requisite for providing patient care in that language. In contrast, applying a gold standard of communicating as native speakers may be in conflict with what is reasonable and feasible. It may result in fewer health professionals demonstrating superior linguistic competency for use in clinical settings, which would lead to even greater reliance on interpreters and subsequent delays in timely access to interpreters for clinical care. A rigorous assessment tool for medical linguistic competency that incoporates the content of most clinical interactions, faculty expertise, patient safety, and medical students’ other pressing educational needs is needed. Such an assessment is relevant for both primary English-speaking medical students seeking to acquire new language skills and students who already have proficiency in a language other than English.

While language curricula are required to build and assess verbal competency in clinical encounters, the hidden curriculum regarding the use of professional versus ad hoc interpreters must also be addressed. The indifference to ensuring appropriate interpreter services for patients with limited English proficiency needs to be rooted out from the clinical learning environment through positive role modeling and facilitating access to qualified interpreters [33]. In one study, one of every 40 malpractice claims were related, all or in part, to failure to provide appropriate interpreter services [34]. Interpreters in health care settings undergo rigorous training and must follow a strict code of ethics [35]. There is a robust literature on the ethics of using professional rather than ad hoc interpreters (e.g. children, family members, friends, and medical assistants who are not qualified interpreters) in medical care. Most importantly, the use of qualified interpreters results in better and more efficient patient care [34, 36, 37].

Recently, the Association of American Medical Colleges (AAMC) published guidelines about core entrustable professional activities (EPAs) that address the foundational behaviors, tasks and responsibilities in which all graduating medical students need to be proficient for clinical care, patient safety, and to enter residency programs [38]. Interpersonal and communication skills are integrated throughout all of the EPAs, yet there is no explicit guidance around appropriate use of interpreters for patients with limited English proficiency in the AAMC EPA Toolkit [39]. Through these EPAs, there is an opportunity to teach appropriate use of interpreters that challenge the predominant hidden curriculum around language services during every clinical encounter.

## Conclusions

We call on medical schools to increase opportunities for medical language courses with evidence-based teaching strategies, integrated content around health equity, and standardized language assessments. The languages offered should reflect the needs of the patient population both where the medical school is located and where the school is engaged globally. Key content areas should include how to conduct a history and physical exam, relevant health inequities that commonly affect the patients who speak different languages, cultural sensitivity and humility particularly around beliefs and practices that affect health and wellbeing, and how to work in language-discordant encounters with interpreters and other modalities. The pedagogical approaches may vary based on the size of the class and resources, but priority should be placed on interactions with health practitioners, patients and community members who are native speakers and immersion opportunities in clinical and community sites when possible. Rigorous language assessment is necessary to ensure equity in communication before allowing students or physicians to use their language skills in clinical encounters. Lastly, global health activities in medical schools should assess for language needs and competency (e.g. local languages and dialects, student fluency in those languages and dialects, plan for language-discordant situations, and availability of interpreters) prior to departure. By professionalizing language competency in medical schools, we can improve patients’ trust in individual physicians and the profession as a whole; improve patient safety and health outcomes; and advance health equity for those we care for and collaborate with in the U.S. and around the world.

## Data Availability

Not applicable.

## Abbreviations

AAMC: Association of American Medical Colleges
ACOs: Accountable Care Organizations
AHCs: Accountable Health Communities
EPAs: Entrustable professional activities
WHO: World Health Organization
